# Intellectual disability health content within medical curriculum: an audit of what our future doctors are taught

**DOI:** 10.1186/s12909-016-0625-1

**Published:** 2016-04-11

**Authors:** Julian N. Trollor, Beth Ruffell, Jane Tracy, Jennifer J. Torr, Seeta Durvasula, Teresa Iacono, Claire Eagleson, Nicolas Lennox

**Affiliations:** Department of Developmental Disability Neuropsychiatry (3DN), UNSW Australia, 34 Botany Street, Randwick, NSW 2052 Australia; Centre for Developmental Disability Health Victoria (CDDHV), Monash Health, 122 Thomas Street, Dandenong, VIC 3175 Australia; Faculty of Medicine, Nursing and Health Sciences, Monash University, VIC, 3800 Australia; Centre for Disability Studies, Sydney Medical School, The University of Sydney, Level 1, Medical Foundation Building, 92-94 Parramatta Road, Camperdown, NSW 2050 Australia; La Trobe Rural Health School, La Trobe University, PO Box 199, Bendigo, VIC 3552 Australia; Queensland Centre for Intellectual and Developmental Disability (QCIDD), Mater Research Institute, The University of Queensland, Level 2 Aubigny Place, Mater Hospitals, South Brisbane, QLD 4101 Australia

**Keywords:** Intellectual Disability, Medical Education, Medical training, Curriculum, Health inequalities

## Abstract

**Background:**

There is a high burden of unmet health needs for people with intellectual disability. Despite experiencing significantly higher rates of morbidity and mortality compared with the general population, this group faces greater barriers to accessing healthcare. While increasing workplace capacity is one way to reduce this inequitable access, previous research indicates a scarcity of undergraduate teaching in intellectual disability. The aim of the study was to determine the extent and nature of intellectual disability content currently offered within medical degree curricula.

**Methods:**

All Australian universities (*n* = 20) providing accredited medical training were invited to participate in a two-phase audit via an email invitation to the Dean of each medical school. The Dean’s delegate from 14 medical schools completed Phase 1, which involved a questionnaire or telephone interview about the overall medical course structure. Unit coordinators and/or teaching staff from 12 medical schools completed Phase 2, which involved an online survey about intellectual disability content within the curriculum.

**Results:**

In Australia, medical school curricula contain a median of 2.55 h of compulsory intellectual disability content. The majority of universities only offer a small amount of compulsory content. Of compulsory units, intellectual disability teaching is minimal in sexual health and emergency medicine (only one unit offered in one school for each). Topics of key relevance in intellectual disability health such as human rights issues, interdisciplinary team work and preventative health are poorly represented in intellectual disability teaching. Elective content varies markedly across universities (1 to 122 h), but emergency medicine, women’s health, men’s health and many other specialist medicine areas are not represented. Inclusive practice is inconsistent in degree and nature, but a majority of universities (nine) involve people with intellectual disability in the development or delivery of content.

**Conclusions:**

There is a mismatch between the considerable unmet health needs of people with intellectual disability and the inconsistent teaching within medical schools. Future doctors will be better equipped to support the health and wellbeing of people with intellectual disability if curricula are enhanced in this area.

**Electronic supplementary material:**

The online version of this article (doi:10.1186/s12909-016-0625-1) contains supplementary material, which is available to authorized users.

## Background

### Health needs and inequalities for people with intellectual disability

People with intellectual disability comprise approximately 1–3 % of the population [1] and compared to the general population, experience significant health disadvantage and poor health outcomes [2], as evidenced by higher morbidity for physical [3] and mental [4] health conditions, and premature death from preventable causes [5, 6]. The burden of unmet health needs in this group is high [3, 7–10], and conditions are frequently undiagnosed or inappropriately treated [11]. Despite living in the general community and being dependent on mainstream health services, people with intellectual disability continue to face disparities in access to preventive healthcare, health promotion and general healthcare [11].

The human rights of individuals with intellectual disability are protected through international agreements, including the United Nations Convention on the Rights of Persons with Disabilities (UNCRPD) [12], to which Australia became a signatory in 2007. Article 25 of this convention obliges signatory states to ensure that persons with disabilities have the right to the highest attainable standard of health without discrimination on the basis of disability. National discrimination acts such as The Equality Act 2010 [13] in the United Kingdom (UK), the Americans with Disabilities Act of 1990 [14] and the Disability Discrimination Act in Australia [15] require government and the community to take action to ensure there is no discrimination on the grounds of disability. Reasonable adjustments must therefore be made to accommodate needs, and to ensure equitable access to services. However, health service providers may lack the knowledge, skills or insights required to make such adjustments.

Initiatives to address the health needs and service gaps experienced by this group include the development of special interest clinical groups [16–21] and academic units. In Australia, the National Disability Strategy [22] has set a clear priority to obtain the highest possible health and well-being outcomes for people with disabilities through the universal equipping of health practitioners and services. Further, the National Disability Insurance Scheme [23] offers the opportunity for disability services to re-design their interface with health services and strengthen pathways to improve access for all Australians with disabilities, including intellectual disability. Similar schemes exist in the UK, with the Direct Payments and Personal Budgets scheme [24], and in Canada, with Self-Managed Care schemes [25].

### What are medical students taught about intellectual disability?

Few studies have documented what student doctors are taught about intellectual disability. The results of one survey of disability and rehabilitation teaching across 23 UK medical schools, published in 1994, indicated that teaching was disjointed: there was little integration across disciplines, and few schools had clear aims [26]. Most of the content taught included medical aspects of disability and rehabilitation. Few schools included ethical aspects, or the role of support services in their curricula. In Malaysia, new medical school graduates were surveyed from seven public hospitals about the education they received on developmental disability (which includes intellectual disability) during undergraduate study [27]. The majority of respondents had studied in Malaysia (71 %), but students who attended medical school abroad were also surveyed. It was found that lecture content in developmental disability was equally apportioned across paediatric and psychiatric curricula. Less information was received in family medicine and public health. While most respondents had contact with people with developmental disabilities while *observing* clinical activities (e.g. giving management advice, surgical intervention), few had the opportunity for direct clinical practice with patients with developmental disabilities. Graduates’ responses also suggested that developmental disability content was less consistently taught to those trained in Eastern and Middle Eastern countries, compared with Malaysia and Western countries. Seven years after the Disability Discrimination Act [15], Lennox and Diggens [28] found major inconsistencies in Australian medical curricula in intellectual disability across universities, and highlighted gaps in relation to relevant skills and attitudes of trainees [29].

One fundamental way to address the unmet needs of people with intellectual disability is to focus on improving the intellectual disability education medical students receive. While surveys have indicated that clinicians lack confidence in the area of intellectual disability health [30, 31], research has demonstrated that medical training that includes intellectual disability content, especially in the form of opportunity for direct contact with people from this population (inclusive teaching), can have a positive effect on trainee confidence, competence and attitude [32–34]. Uniform provision of intellectual disability content in medical curricula is desirable as it will encourage the development of mainstream capacity of primary health care providers and their services, and will equip practitioners with much needed communication, assessment and management strategies for patients [35, 36]. This can also help to meet human rights and legislation requirements that the community takes steps to ensure no discrimination on the basis of disability [12, 15]. Evidence suggests that tertiary education is the best time to influence the skills, knowledge, and attitudes of health professionals [37, 38]. Further, teaching in speciality areas is associated with a greater number of graduates choosing to work in those areas [39]. As specialist pathways in intellectual disability health and mental health are available in paediatrics and psychiatry respectively, the inclusion of intellectual disability content may encourage some to pursue this area as a speciality interest. This would provide a much needed boost to the availability of specialist health care for people with intellectual disability. Increasing the coverage of intellectual disability during undergraduate medical education could go a considerable way to addressing the unmet health needs of this population by improving workforce capacity.

The development of clinical skills in intellectual disability health has relevance beyond this specific clinical area. Apart from specific content knowledge, key skills for effective clinical practice in this area include the ability to adapt the clinical consultation to the patient’s level of ability, adjust communication, work with individuals with multiple disabilities, work effectively in a multidisciplinary framework, support decision making capacities, and work with families and carers [40]. Thus teaching medical students about intellectual disability health has relevance to a much larger group of individuals including those with other developmental and acquired cognitive disorders.

Despite these findings and arguments, there is no requirement for medical programmes to include teaching content specific to intellectual disability to meet accreditation requirements [41]. This lack of requirement is also evident in other countries, such as the United States, where the standards for medical school program accreditation do not include stipulation of specific areas, such as intellectual disability, but rather refer to broader curriculum content that can apply across multiple areas (e.g. medical ethics) [42].

### The current study

Despite previous research demonstrating a paucity of undergraduate medical teaching in intellectual disability [26, 28, 29], the current status of medical curricula is not known. We conducted a national audit of medical curricula across Australian medical schools to assess content in relation to intellectual disability. The current project is the first of a multistage strategy to build capacity within the health workforce in Australia by renewing the medical and nursing intellectual disability curriculum. In turn, it is also hoped that this research can inform development of the health workforce internationally.

## Methods

**Fig. 1:**
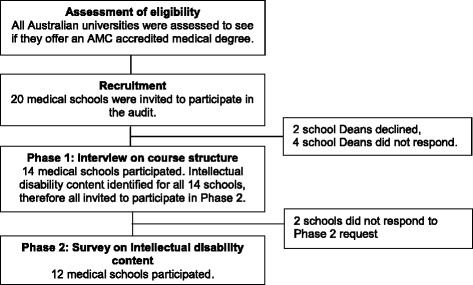
Recruitment and participation rates

A two phase national audit of medical curricula content was conducted in 2013–14. Figure 1 shows details of the recruitment and data collection procedures. The Deans of the 20 medical schools that deliver Australian Medical Council (AMC) accredited medical degrees in Australia were approached via email and invited to participate. Permission to participate was granted by fourteen medical schools. The Dean’s representative then completed an interview on the medical course structure and identified potential course components where intellectual disability may reside, and the relevant coordinators of these course units (Phase 1). An email was then sent to each identified coordinator to complete a survey regarding the content of the unit(s) (Phase 2). Survey responses for Phase 2 were obtained from twelve ‘participating medical schools’ and this formed the basis of the results for the current study (two schools did not respond to the Phase 2 request). All participants read a participant information sheet before taking part and completion of the interview or survey was taken as implied consent. A protocol of three email reminders and a telephone call was followed at each phase. Institutions were coded to preserve anonymity during data analysis and reporting.

For enhanced comparability of audit results, the majority of questions were the same as those used by Lennox and Diggens [28]. However, we included additional questions to obtain more detail regarding broad categories, discipline and topic areas of medicine covered by intellectual disability content. See Table 1 for details of question domains and categories. In Phase 1, 11 interview questions on course structure were answered over the telephone or completed on written proforma and returned by email (see Additional file 1). In Phase 2, 16 questions on specific intellectual disability content were answered via an online survey (see Additional file 2 for detailed survey tool). Ethical approval for the study was obtained from the UNSW Australia Human Research Ethics Advisory Panel (Approval No. 2013-7-03).

**Table 1:** Question domains and categories within domains

	Domain	Question category
Phase 1	Course structure	Program type; total units; entry level; duration; number of students; contact hours; number of compulsory units; number of elective units; number of units containing intellectual disability specific content.
	School staff profile	Total number of staff specialising in intellectual disability; total number staff with a demonstrated interest in intellectual disability; total number of staff who teach intellectual disability content.
Phase 2	Unit details	Year of course; compulsory or elective enrolment; total number of students enrolled.
	Intellectual disability content	Total intellectual disability teaching time; type of intellectual disability content; topics covered; subject area of medicine; direct clinical contact.
	Teaching style	Teaching mode; inclusion of people with intellectual disability in the development or delivery of content; assessments; learning style.
	Teaching staff profile	Professional background; university staff, conjoint or external employment.

## Results

### Course programmes and length

Of the twelve medical schools that participated in Phase 2, eleven offered a Bachelor of Medicine, Bachelor of Surgery (MBBS) and one a Doctor of Medicine (MD) programme. Five participating schools offered undergraduate entry only, five offered graduate entry only, and two offered both entry pathways. Six schools offered a 4-year course, two offered a 5-year course, and four offered a 6-year course. The number of students enrolled varied across institutions (range = 99–520, median = 182).

### Intellectual disability units

‘Intellectual disability units’ refers to discrete course components containing some auditable content specific to intellectual disability.

#### Compulsory units

**Fig. 2:**
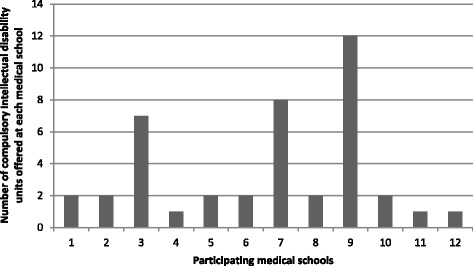
Total number of compulsory intellectual disability units offered by participating universities

The number of compulsory intellectual disability units offered across participating medical schools is presented in Fig. 2. As shown, the 12 participating schools provided a total of 42 compulsory intellectual disability units. A small number of medical schools (3 schools, 25 %) provided the majority of units (27 units, 64 %). The total time dedicated to teaching intellectual disability content varied (range = 30 min–18 h, median = 2.55 h). Seven participating schools (60 %) provided less than a total of six hours of teaching time on intellectual disability.

#### Elective units

Six participating medical schools offered a total of eight elective intellectual disability units (range = 1–3, median = 1). The number of students enrolled in these elective units varied (range = 3–180, median = 12.5), the total time spent teaching intellectual disability content in elective units ranged from 1 to 222 h (median = 3 h). A single unit offered by one participating school included 222 h of intellectual disability content, with eight students (2 % of the student population) enrolled in 2013/14.

### Distribution of intellectual disability units and enrolment

**Fig. 3:**
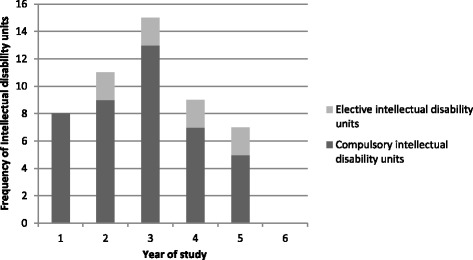
Distribution of compulsory and elective intellectual disability content in medical courses

Intellectual disability content was unevenly distributed across years of study (Fig. 3), with more units offered in the third year than any other year.

### Compulsory intellectual disability content

#### Broad categories

Nine medical schools provided compulsory intellectual disability content that included both physical and mental health. Two schools provided compulsory content that covered only physical health, while one provided compulsory content that covered only mental health.

#### Discipline areas

Table 2 provides details about the discipline areas covered within compulsory intellectual disability units. Those most consistently covered included paediatrics, general practice and psychiatry, and those least covered included emergency medicine and sexual health.

**Table 2:** Discipline areas of medicine and specific topic areas covered within compulsory intellectual disability content

	Schools *N*	Units *N*
Discipline Area^a^		
Paediatrics	9	11
Other^b^	6	7
General Practice	4	7
Psychiatry	3	5
Women’s health	2	2
Other specialist medicine^c^	1	8
Sexual health	1	1
Emergency Medicine	1	1
Topic Area^d^		
Clinical assessment skills	9	27
Clinical management skills	9	21
Ethics and legal issues	8	20
Chronic and complex health issues	8	17
Interdisciplinary team work	8	12
Preventative health	7	10
Disability and health care systems	6	20
Human rights issues	6	16

^a^Six compulsory units were offered which covered two or more discipline areas

^b^Includes: Disability studies, human development, professional development, societal aspects of intellectual disability

^c^Includes: cardiovascular, endocrinology, genetics, head and neck, musculoskeletal, neurosciences, obstetrics, rehabilitation

^d^28 units were offered which covered two or more topic areas

N.B. Data from one school was unavailable

#### Specific topic areas

Table 2 also provides details of specific topic areas included within compulsory intellectual disability units. Those most consistently covered included clinical assessment and management skills, and those least covered included human rights, and disability and health care systems.

### Elective intellectual disability content

For elective intellectual disability content, two medical schools provided content in both physical and mental health, two schools provided content in physical health only and two in mental health only. Elective content in the areas of paediatrics and professional development was offered at two schools and in general practice and sexual health at one school only. In no school was elective intellectual disability content offered in the areas of emergency medicine, women’s health, men’s health or other specialist areas of medicine (e.g. cardiovascular). The most frequently represented elective topic (6 participating schools) was interdisciplinary team work. The elective topics of clinical management skills, disability and health care systems, and human rights were each offered at four participating schools.

### Inclusive practice in intellectual disability education

Compulsory intellectual disability units involving direct clinical contact with people with an intellectual disability were offered in only five medical schools (42 %). Such contact occurred within inpatient facilities, specialist clinics and community health settings, but not within disability accommodation, residential or educational settings (see Table 3). People with intellectual disability had been directly involved in the development or delivery of compulsory content in seven (58 %) medical schools.

**Table 3:** Direct clinical contact environment offered in intellectual disability units

Clinical environment^a^	Compulsory		Elective	
	Schools *N*	Units *N*	Schools *N*	Units *N*
Inpatient facility	4	6	1	1
Specialist clinic	4	5	1	3
Community health setting	4	5	1	1
Disability service	2	2	1	1
General practice	1	1	2	2
School	1	1	0	0
Group/family home	0	0	2	2

^a^Five compulsory units and four elective units included contact in two or more clinical environments

Elective intellectual disability content involving direct clinical contact with people with an intellectual disability was offered in two (17 %) medical schools. People with intellectual disability had been directly involved in the development or delivery of elective content in three (25 %) medical schools.

## Discussion

### Overview of findings

The audit revealed that all participating universities provided some compulsory intellectual disability content. However, the amount of content varied greatly between institutions, with the majority of responding institutions offering only a small amount of compulsory content. Overall, the time spent teaching compulsory and elective intellectual disability content was minimal, with the majority of schools offering less than six hours of compulsory teaching time across the entire course. Key topic areas of relevance to the health and well-being of people with intellectual disability were taught infrequently. Despite what is known about psychiatric comorbidities [4] and primary health care needs [11] for people with intellectual disability, only a third of participating schools provided compulsory content in these areas. Similarly, despite this group’s high unmet health needs [3, 7–10] and significant health inequalities [2, 43], only half of participating schools provided compulsory teaching relating to human rights issues, and disability and health care systems. Elective intellectual disability content in mental and/or physical health was only taught by half of the participating schools. Where provided, elective content was inconsistent in nature, and varied greatly in duration and number of student enrolments.

### Comparison with past audits

The current findings are similar to that of Lennox and Diggens [28] who also found inconsistencies across medical curricula in the degree to which intellectual disability content was taught. They found that in some cases, teaching in intellectual disability was integrated across the curriculum, whilst in others, a small amount of content in limited areas was offered [28]. Their audit found that 70 % of schools included compulsory teaching involving direct clinical contact with people with intellectual disability, a higher figure than obtained in the current audit. A positive finding, however, was that inclusive teaching practices in compulsory units have increased from 30 % of schools in 1999 [28] to 58 % (7 schools) in the current audit.

The inconsistencies found in intellectual disability teaching across universities also mirrored results from the 1994 UK audit [26]. Parallel to the current study’s finding that clinical assessment and management skills were most often taught, with little focus on disability and health care systems, the majority of content taught in the UK medical schools was medical aspects of disability, with minimal teaching on support services. Medical graduates working in Malaysian public hospitals reported receiving most of their developmental disability education across paediatric and psychiatric curricula in comparison with other areas [27]. However we found the majority of compulsory units were offered in paediatrics, while only five units were in psychiatry.

### Current curricula in the context of research and policy

This audit suggests that current teaching about intellectual disability in medical schools will not address the recognised lack of clinician confidence in this area [30, 31], will not encourage the development of future workforce capacity in both mainstream and specialised intellectual disability healthcare, nor assist Australia to meet human rights [12] and anti-discrimination [15] requirements. Signatory states of the UNCRPD [12] have an obligation to meet the requirements outlined in the Convention, which means systemic change in the attitudes and values of practitioners, in health systems and in clinical skills including assessment, diagnosis, contextualised management and team work. This research suggests that Australia, a signatory state, does not have a coherent approach to the training of medical practitioners to meet the health needs of people with intellectual disability. Where teaching is provided, this has improved, but some areas such as in the provision of direct clinical contact with people with intellectual disability have seen regression.

Although inclusive practice in this area would fulfil human rights legislation [12, 15], implementation of inclusive approaches in medical schools was patchy. For example, although the involvement of people with intellectual disability in the development or delivery of compulsory content was noted for just over half of the participating medical schools, such involvement was far less likely for elective units, and direct clinical contact with people with intellectual disability occurred in a minority of schools. Inclusive teaching, particularly that which involves direct clinical contact with people with intellectual disability is of critical importance as it shapes attitudes, instils confidence and improves competence [32–34] in this clinical territory. Teaching initiatives in this area should therefore be required to include people with intellectual disability as a cornerstone in curriculum development, delivery and during clinical placements.

The clinical skills acquired while learning about intellectual disability, such as alternative communication methods, working in a multidisciplinary environment and involving families and carers can be used to improve clinical practice with other populations such as older adults and those with other disabilities. Such training would therefore be seen as an asset to current curricula, as it would equip students with higher level skills which could be employed in a variety of settings. It is clear there is a multifaceted case for including a greater focus on intellectual disability education in medical schools which in turn can lead to direct benefits for a population that has considerable unmet health needs.

Results from this study should be considered in the light of some key limitations. Engagement of medical schools in the audit may reflect a positive disposition and the presence of relevant content, which may have inflated figures relating to representation of intellectual disability content. Data collection relied heavily on email communication, which although aimed to reduce the burden on participating staff, may have increased fatigue and reduced engagement. As data were collected remotely, it was not possible to determine whether the information provided was comprehensive. Some of the questions were open-ended, so there was the possibility that respondents may have interpreted the questions in slightly different ways. For questions that were forced choice, the choices may not have covered all possible categories of response. Further, inconsistency in course structures and definitions of individual units of study made it difficult to directly compare the proportion of curriculum dedicated to specific intellectual disability teaching across schools.

In relation to the current findings, we recommend that medical schools respond to the evident health need and human rights legislation to ensure the training of medical professionals is comprehensive, principle based and consistent, with respect to the healthcare needs of children and adults with intellectual disability. There are plans to develop, evaluate and implement a national education framework and implementation toolkit for medical schools, which will provide up to date, evidence-based teaching materials and resources so universities can develop a comprehensive intellectual disability curriculum.

## Conclusions

The current study was the first national audit of intellectual disability content in medical curricula to be conducted in Australia in 15 years. Our findings suggest that current intellectual disability content taught within medical training in Australia is highly variable and remains limited. Although more medical schools are now including some education in this area, there has been little movement towards a consistent and comprehensive medical curriculum in intellectual disability health, despite clear evidence of health disadvantage, recommendations from previous research [26, 28], significant changes in international and national human rights legislation and requirements for equity in service access [12, 15]. Research has shown that inclusion of such content can improve attitudes and better prepare trainees to work with this population [32–34], provide skills which can enhance mainstream health services for people with intellectual disability [35, 36], and will likely encourage more graduates to specialise in this area [39]. There is therefore a well-defined and evidence-based need to improve medical training in this area, in order to build a workforce which is better equipped to meet the complex health needs of this population, and to improve equity of access to healthcare. With a greater emphasis on intellectual disability education, graduating medical practitioners will be better equipped to recognise and address current health disparities, and provide high quality healthcare in order to improve health outcomes for people with intellectual disability.

## Ethics approval and consent to participate

Ethical approval for the study was obtained from the UNSW Australia Human Research Ethics Advisory Panel (Approval No. 2013-7-03). All participants read a participant information sheet before taking part and completion of the interview or survey was taken as implied consent.

## Consent for publication

Not applicable.

## Availability of data and materials

Please contact the first author for data and materials used in this study.

## Additional files

Additional file 1: Phase 1: Interview Schedule- relating to the overall structure of the course. (PDF 172 kb) Additional file 2: Phase 2: Survey Schedule- relating to specific unit of study. (PDF 182 kb)

## Abbreviations

AMC: Australian Medical Council
MBBS: Bachelor of Medicine, Bachelor of Surgery
MD: Doctor of Medicine programme
UNCRPD: United Nations Convention on the Rights of Persons with Disabilities
