# Erratum to: Intellectual disability health content within medical curriculum: an audit of what our future doctors are taught

**DOI:** 10.1186/s12909-016-0784-0

**Published:** 2016-10-05

**Authors:** Julian N. Trollor, Beth Ruffell, Jane Tracy, Jennifer J. Torr, Seeta Durvasula, Teresa Iacono, Claire Eagleson, Nicolas Lennox

**Affiliations:** 1 Department of Developmental Disability Neuropsychiatry (3DN), UNSW Australia, 34 Botany Street, Randwick, 2052 NSW Australia; 2 Centre for Developmental Disability Health Victoria (CDDHV), Monash Health, 122 Thomas Street, Dandenong, 3175 VIC Australia; 3 Faculty of Medicine, Nursing and Health Sciences, Monash University, Melbourne, 3800 VIC Australia; 4 Centre for Disability Studies, Sydney Medical School, The University of Sydney, Level 1, Medical Foundation Building, 92-94 Parramatta Road, Camperdown, 2050 NSW Australia; 5 La Trobe Rural Health School, La Trobe University, PO Box 199, Bendigo, 3552 VIC Australia; 6 Queensland Centre for Intellectual and Developmental Disability (QCIDD), Mater Research Institute, The University of Queensland, Level 2 Aubigny Place, Mater Hospitals, South Brisbane, QLD 4101 Australia

## Erratum

After publication of the original article [1], the authors found two errors within the Results section of the Abstract. Details are as follows:

1. In the line ‘Elective content varies markedly across universities (1 to 122 h)’, the number “122” should be “222”.
2. In the line ‘In Australia, medical school curricula contain a median of 2.55 h of compulsory intellectual disability content’, there should be a word “units” inserted between “curricula” and “contain”. The full corrected sentence should read: ‘In Australia, medical school curricula units contain a median of 2.55 h of compulsory intellectual disability content’.
