# Supplementary material for: Intellectual disability health content within medical curriculum: an audit of what our future doctors are taught
Source: BMC Med Educ. 2016 Apr 11;16:105. doi: 10.1186/s12909-016-0625-1 (PMC4827238; doi:10.1186/s12909-016-0625-1)
Supplement: Additional file 1: — Phase 1: Interview Schedule- relating to the overall structure of the course. (PDF 172 kb) [file 12909_2016_625_MOESM1_ESM.pdf]

Additional file 1. Phase 1: Interview Schedule- relating to the overall structure of the course

1) What is the name of your University?

2) What is your current position within the University?

3) What is the name of the medical degree offered by your university?

4) Is this degree graduate entry level or undergraduate?

5) What is the duration of the degree for a full-time student (in years)?

6) How many students graduated from the degree in the last academic year?

7) Approximately what percentage of students' time during the degree involves direct patient contact?

8) In total, how many subjects/units of study (including both non-clinical and clinical units) are provided as part of this degree?

9) Of the total subjects/units of study in the degree:

   a. How many are compulsory subjects/units of study?
   b. How many are elective subjects/units of study?
      i. Of the elective subjects/units of study do any of them have an intellectual disability component?
      ii. If yes, approximately what proportion of students choose this intellectual disability component?

10) How many subjects/units of study (including both non-clinical and clinical units) in this degree contain at least some formal teaching content in intellectual disability physical health and/or intellectual disability mental health?

11) How many staff within the medical school:

   a. Specialise in the area of intellectual disability physical health and/or intellectual disability mental health?
   b. Have a demonstrated interest in the area of intellectual disability physical health and/or intellectual disability mental health?
   c. Currently provide teaching within the curriculum specifically in the area of intellectual disability physical health and/or intellectual disability mental health?
