# Supplementary material for: Intellectual disability health content within medical curriculum: an audit of what our future doctors are taught
Source: BMC Med Educ. 2016 Apr 11;16:105. doi: 10.1186/s12909-016-0625-1 (PMC4827238; doi:10.1186/s12909-016-0625-1)
Supplement: Additional file 2: — Phase 2: Survey Schedule- relating to specific unit of study. (PDF 182 kb) [file 12909_2016_625_MOESM2_ESM.pdf]

Additional file 2. Phase 2: Survey Schedule- relating to specific unit of study

1. What year is this unit offered?

2. How many hours of direct teaching or clinical placement are involved in this unit of study?

3. Is it compulsory or elective?

4. How many students are enrolled in this unit of study?

5. Does the unit involve direct patient contact?

6. Does the unit include formal teaching in intellectual disability physical health and/or intellectual disability mental health?
    a. If yes, how many hours?

7. What area of medicine?
    a. General practice
    b. Paediatrics/developmental paediatrics
    c. Psychiatry
    d. Emergency Medicine
    e. Other______

8. Mode of delivery?
    a. Lecture
    b. Tutorial
    c. Workshop
    d. Other______

9. Clinical contact environment:
    a. Inpatient
    b. Community
    c. General practice
    d. Specialist clinic
    e. Disability service
    f. Group home
    g. Family home of a person with an intellectual disability
    h. School
    i. Other______

10. Do the assessments involve specific intellectual disability content (mental health or physical health)

11. What style of learning?
    a. Problem-based learning
    b. Enquiry based learning

12. Who teaches the intellectual disability component?
    a. University staff
    b. External staff
    c. Conjoint appointees
    d. Other_________

13. What is their professional background?
    a. Psychiatrist
    b. Doctor
    c. Psychologist
    d. Registered Nurse
    e. Allied Health Professional
    f. Other______

14. Does the intellectual disability content cover:
    a. Clinical assessment skills
    b. Clinical management skills
    c. Ethics and legal issues
    d. Preventative health
    e. Disability and health care service systems
    f. Interdisciplinary team work
    g. Human rights issues in disability
    h. Chronic and complex health issues

15. Are people with intellectual disability involved in the development and/or delivery of this study?
    a. If yes, how are they involved?

16. Provide an overall description of the intellectual disability physical health and/or intellectual disability mental health content in this unit of study
